# Root and arch replacements in acute type A aortic dissection suggest lower risk of open distal reintervention

**DOI:** 10.1016/j.xjse.2025.100049

**Published:** 2025-03-26

**Authors:** Bo Chang Brian Wu, Adam M. Carroll, Nicolas Chanes, Michal Schafer, Tylor Thai, R. Wilson King, Zihan Feng, Muhammad Aftab, T. Brett Reece

**Affiliations:** a Department of Surgery, University of Colorado School of Medicine, Aurora, Colo; b Division of Cardiothoracic Surgery, Department of Surgery, University of Colorado School of Medicine, Aurora, Colo; c Division of Cardiothoracic Surgery, Department of Surgery, University of Utah School of Medicine, Salt Lake City, Utah; d Division of Vascular Surgery, Department of Surgery, University of Colorado School of Medicine, Aurora, Colo; e Department of Surgery, University of Pittsburgh, Pittsburgh, Pa

**Keywords:** aortic dissection, arch replacement, reintervention, reoperation, root replacement

## Abstract

**Objective:**

This study aimed to evaluate how different approaches for root and arch management during the first surgery for acute type A aortic dissection impact long-term freedom from reintervention, particularly distal reintervention.

**Methods:**

This is a retrospective cohort study analyzing 164 patients who underwent acute type A aortic dissection surgery from January 2009 to April 2024. Patients were stratified into root replacement (n = 75) and non-root replacement (n = 89) groups. The root replacement group was further stratified by arch intervention type. Kaplan-Meier analysis and Cox regression models were performed to assess the impact of root and arch interventions on freedom from reinterventions.

**Results:**

Root replacement significantly reduced the risk of any reintervention (hazard ratio, 0.496, *P* = .032) and open distal reintervention (hazard ratio, 0.307, *P* = .037) in univariate analyses, but not in multivariate analysis. Total arch replacement significantly reduced the risk of open distal reintervention (hazard ratio, 0.056, *P* = .011) in multivariate analysis. Patients undergoing combined root and total arch replacement demonstrated 100% 5-year freedom from open distal reintervention, compared with 82% for combined root and hemiarch replacement and 76% for non-root replacement, although only a trend was observed (p_logrank_ = .060).

**Conclusions:**

Root and total arch replacements in acute type A aortic dissection demonstrated reduced risk of reinterventions, particularly open reintervention distal to the arch. More aggressive approaches have the potential to reduce the burden and cost of subsequent operations and improve long-term outcomes. However, although root and total arch replacements offer excellent long-term benefits, they are more complex with potentially higher perioperative risks. The decision to pursue more aggressive interventions should be based on a comprehensive patient assessment.

**Figure:**
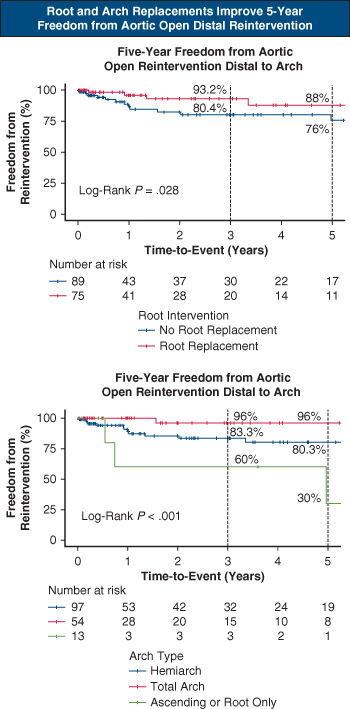
More aggressive root and arch approaches may reduce open reintervention in type A dissection.

**Central Message**

More aggressive root and arch approaches in ATAAD may reduce the risk of open distal reoperation, lowering the burden and cost of subsequent procedures while improving long-term outcomes.

**Perspective**

The long-term impact of different root and arch approaches on distal reintervention remains unclear. This study showed that root and TAR significantly reduced reintervention risk. Combined root and total arch had 100% 5-year freedom from open distal reintervention. More aggressive approaches may lower the burden and cost of subsequent operations, and improve long-term outcomes.

Acute type A aortic dissection (ATAAD) is a devastating and life-threatening condition that remains a challenge to aortic surgeons, although operative mortality has improved from 25% to 18% according to the International Registry of Acute Aortic Dissection.^1^ Modifications and refinements in surgical techniques along with perioperative care have significantly improved short-term morbidity (eg, stroke, malperfusion) and mortality.^2^, ^3^, ^4^ After primary ATAAD repairs, the 10-year risk of distal reoperation is up to 38% after ascending aorta replacement,^5^ but the risk is as low as 15% after hemiarch replacement (HAR).^6^ Another study reports 10-year freedom from any reintervention of 64.2%.^7^ For survivors of ATAAD repair, the 10-year survival is approximately 58% to 66%.^5^^,^^8^^,^^9^ Additionally, total arch replacement (TAR) improves long-term survival compared with ascending replacement.^10^

Debates exist in the choice of root intervention and arch extent.^2^ Current American College of Cardiology/American Heart Association guidelines for acute aortic syndrome management recommend more conservative approaches to valve, root, and arch intervention when feasible.^11^ When repairable, aortic valve resuspension over valve replacement is recommended. In cases of extensively destructed roots, root replacement (RR) with a biological or mechanical valved conduit is preferred. Valve-sparing root repair (VSRR, eg, David procedure) is recommended only when performed by experienced surgeons. Additionally, hemiarch repair is recommended over a more extensive arch replacement in patients without an intimal tear entry point in the arch or a significant aneurysm.^11^ An open distal anastomosis is recommended to improve survival and increase false lumen thrombosis rates. In cases where a dissection flap extends through the arch into the descending thoracic aorta, an extensive approach with antegrade stenting should be considered to treat malperfusion and lower the distal aortic complications.^12^^,^^13^

The management of root repair varies, with surgeons choosing between conserving or replacing the root. Root-sparing operations have been suggested for simplicity, but RR is recommended because of the long-term risks associated with preserving the root. However, this approach carries short-term risks because it is technically more demanding and may increase operative risk.^14^ Current literature compares different approaches and their outcomes, with some focusing on the freedom from proximal aortic reintervention.^14^ It is unclear how root strategy impacts the pathology distal to the aortic arch.

Although HAR is usually favored as the standard approach for arch management, particularly when the primary entry point is in the ascending aorta or lesser curvature of the arch, some advocate for more extensive procedures (eg, TAR). This debate is driven by concerns over future complications, such as distal degeneration, dissection, and persistent false lumen.^15^^,^^16^

The study aimed to evaluate how root and arch management at index surgery for ATAAD impacts freedom from reintervention, particularly reintervention distal to the arch.

## Material and Methods

### Study Design and Data Collection

**Figure 1:**
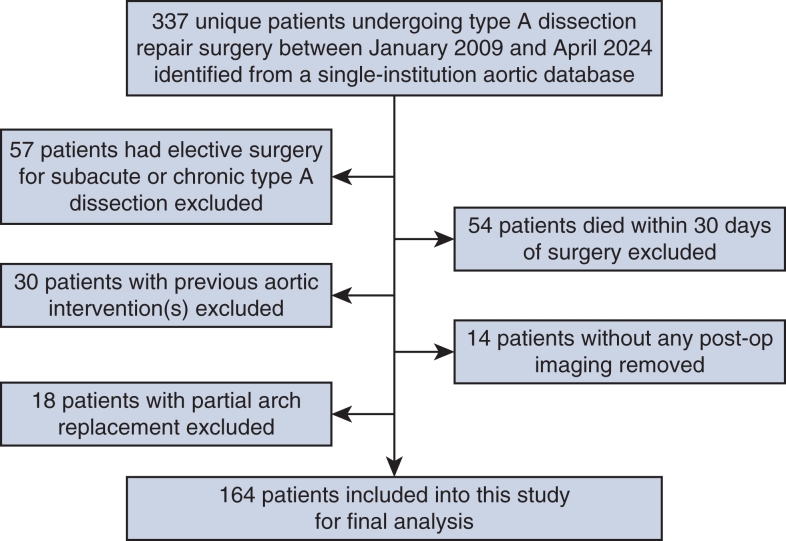
Patient inclusion and exclusion criteria for this study.

This is a retrospective cohort study analyzing a single-institution database. The study design was approved on February 6, 2017, by the Colorado Multiple Institutional Review Board (COMIRB #17-0198), which waived the requirement for informed consent. The cohort included patients presenting with ATAAD from 2009 to April 2024. Of 337 patients reviewed, 164 met the inclusion criteria after excluding 57 patients with elective repair, 54 patients who died within 30 days of surgery, 30 patients with previous aortic intervention(s), 14 patients without postoperative follow-up imaging available, and 18 patients undergoing partial arch replacement (eg, zone 1 or 2 arch replacement) (Figure 1). The study end period was July 2024, with data collection completed in November 2024.

### Patient Categorization

Patients were categorized into 2 primary groups: RR (including Bentall and David procedures) and non-RR (valve resuspension with root repair/remodeling or no root intervention at all). Patients were further stratified by the extent of arch intervention: hemiarch, total arch, and no arch replacements. A subanalysis evaluated combinations of root and arch interventions. Patients with partial arch replacement were excluded to minimize confounding.

### Data and Variables

Preoperative variables included demographics, medical history, urgency status, dissection extent (eg, root, ascending, arch, or descending), preoperative hemodynamic instability, and preoperative aneurysm. Preoperative instability was defined as cardiogenic shock (including tamponade or arrest), hypertensive crisis, need for intubation, requirement for mechanical circulatory support, or presence of malperfusion. Operative variables included aortic valve, root, ascending, arch interventions, and antegrade thoracic endovascular stenting. Postoperative outcomes were imaging stability over time (up to ≥18 months postoperatively), late mortality status (>30 days postoperatively), time to late death, and aortic reintervention locations (eg, any reintervention, aortic valve reintervention, root reintervention, arch reintervention, reintervention distal to the arch—distal reintervention, including descending aorta and interventions with pure thoracic endovascular aortic repair [TEVAR]), and time to aortic reintervention. Open distal reintervention included frozen elephant trunk (FET) and other open interventions. Additionally, time to last follow-up via computed tomography imaging or cardiac surgery visit (if no reintervention) and rates of loss to follow-up at various time points were analyzed. Regarding imaging stability, if patients did not have an imaging performed or available at the specified time points, those data were marked as not available, and only the available data were included in the analysis. Two follow-up indices were calculated.^17^ The first was based on imaging and cardiac surgery visits, with reoperation as a censoring event. The second was based on survival, with death as a censoring event. High-volume surgeons, defined as performing 10 type A or more dissection repairs per year regardless of urgency and redo status, were assessed for their impact on reintervention risk. This study included cases performed by 10 surgeons, with 1 consistently maintaining a high volume with 13.4 cases per year.

### Criteria for Root and Arch Replacement

RR was performed for dissection extending into the root or root aneurysm. Limited dissection could be managed with root repair. The extent of arch replacement was determined by factors such as the extent of dissection, distal malperfusion, and computed tomography imaging showing a true lumen diameter less than 20% of aortic size.

### Statistical Analysis

R software (version 4.4.1) was used for statistical analyses. Comparative statistical methods included *t* tests, analysis of variance, Mann–Whitney *U*, Kruskal–Wallis, chi-square, and Fisher exact tests. Propensity score matching was used for age, gender, and preoperative aneurysm status to compare outcomes between root and non-root replacements. Kaplan–Meier analysis was performed to assess reintervention, with curves truncated at 5 years when the number at risk in each subgroup was approximately 10% or greater. Cox proportional-hazard models evaluated the risk of reintervention.

## Results

### Patient Characteristics, Operative Approaches, and Postoperative Outcomes

Among 164 patients (mean age 59.2 ± 13.0 years, 68.9% male, mean body mass index [BMI] 28.6 ± 5.4), 78.7% had hypertension. Additionally, 30.5% had a smoking history and 3.0% had connective tissue disorder. Most underwent emergency operations (81.7%). Operative approaches included aortic valve replacement (AVR) in 43.9% of patients, RR in 45.7%, ascending replacement in 95.1%, HAR in 59.1%, and TAR in 32.9%. Regarding imaging stability, 87.2% had stable imaging at 1 month postoperatively, 93.8% at 3 months, 94.1% at 6 months, 82.5% at 1 year, and 80% at more than 18 months. The average time to first postoperative imaging was 36 ± 54.4 days. Mean time to reoperation was 1.7 ± 2.6 years, and mean time to late death was 3.3 ± 3.1 years. The overall aortic reoperation rate was 28.7%, with reintervention rates of 11.6% at the root, 14% at the arch, and 22.6% distal to the arch. Late mortality was 7.9%. Mean follow-up indices were 60% (imaging and visits) and 70% (survival based).

### Root Replacement Versus Non-Root Replacement

Compared with the non-root replacement group (non-RR, n = 89), the root replacement group (RR, n = 75) was older (56.4 ± 12.9 vs 61.5 ± 12.7 years, *P =* .011) and had more male patients (77.3% vs 61.8%, *P =* .042), more aneurysms (42.7% vs 24.7%, *P =* .019), and more dissection involving root (81.3% vs 34.8%, *P <* .001) (Table 1). No significant differences were observed in BMI, other medical history, urgency status, and preoperative hemodynamic stability. AVR was performed more frequently in RR (86.7% vs 7.9%, *P <* .001). Other operative approaches were similar between the 2 groups. Imaging stability over time, time to first postoperative imaging, time to first reoperation, and time to death showed no significant differences.

**Table 1:** Comparisons of preoperative, operative, and postoperative variables between root and non-root replacements

Variable	Root replacement (N = 75)	Non-root replacement (N = 89)	Overall (N = 164)	*P* value
Patient demographics and comorbidities				
Age, y	56.4 ± 12.9	61.5 ± 12.7	59.2 ± 13	**.011**
Sex (male)	58 (77.3%)	55 (61.8%)	113 (68.9%)	**.042**
BMI	28.7 ± 5.4	28.5 ± 5.5	28.6 ± 5.4	.775
Hypertension	59 (78.7%)	70 (78.7%)	129 (78.7%)	1.000
Smoking	20 (26.7%)	30 (33.7%)	50 (30.5%)	.395
Connective tissue disorder	3 (4%)	2 (2.2%)	5 (3%)	.661
Presentation				
Urgency status (emergency)	63 (84%)	71 (79.8%)	134 (81.7%)	.547
Extent of dissection	-	-	-	-
Root	61 (81.3%)	31 (34.8%)	92 (56.1%)	**<.001**
Ascending	69 (92%)	82 (92.1%)	151 (92.1%)	1.000
Arch	52 (69.3%)	72 (80.9%)	124 (75.6%)	.102
Descending	32 (42.7%)	34 (38.2%)	66 (40.2%)	.632
Preoperative hemodynamic instability	29 (38.7%)	30 (33.7%)	59 (36%)	.519
Cardiogenic shock	13 (17.3%)	8 (9%)	21 (12.8%)	.159
Hypertensive crisis	2 (2.7%)	4 (4.5%)	6 (3.7%)	.689
Massive transfusion protocol	0 (0.0%)	0 (0.0%)	0 (0%)	1.000
Intubation	3 (4%)	2 (2.2%)	5 (3%)	.661
Mechanical circulatory support	0 (0.0%)	1 (1.1%)	1 (0.6%)	1.000
Malperfusion	18 (24%)	22 (24.7%)	40 (24.4%)	1.000
Aneurysm	32 (42.7%)	22 (24.7%)	54 (32.9%)	**.019**
Other operative interventions				
AVR	65 (86.7%)	7 (7.9%)	72 (43.9%)	**<.001**
Ascending	69 (92%)	87 (97.8%)	156 (95.1%)	.144
Hemiarch	46 (61.3%)	51 (57.3%)	97 (59.1%)	.635
Total arch	28 (37.3%)	26 (29.2%)	54 (32.9%)	.318
TEVAR	32 (42.7%)	32 (36%)	64 (39%)	.424
Postoperative outcomes				
Time from OR to death (y)	4.3 ± 4.6	2.7 ± 2.0	3.3 ± 3.1	.724
Late death (beyond 30 d postoperatively)	5 (6.7%)	8 (9%)	13 (7.9%)	.773
Stable ≥18-mo postoperative imaging	29 (90.6%)	35 (72.9%)	64 (80%)	.085
Time to last follow-up or imaging if no surgical intervention (y)	2.3 ± 2.6	3.4 ± 3.6	2.8 ± 3.2	.191
Time to first reoperation (y)	0.9 ± 1.3	2.1 ± 2.9	1.7 ± 2.6	.171
Reoperation	13 (17.3%)	34 (38.2%)	47 (28.7%)	**.003**
Valve	1 (1.4%)	15 (16.9%)	16 (9.8%)	**.001**
Root	3 (4%)	16 (18%)	19 (11.6%)	**.006**
Ascending	4 (5.3%)	16 (18%)	20 (12.2%)	**.016**
Distal to root	11 (14.7%)	28 (31.5%)	39 (23.8%)	**.016**
Arch	5 (6.7%)	18 (20.2%)	23 (14%)	**.014**
Distal to arch	11 (14.7%)	26 (29.2%)	37 (22.6%)	**.038**
Distal to arch (open)	4 (5.3%)	16 (18%)	20 (12.2%)	**.016**
Descending	11 (14.7%)	26 (29.2%)	37 (22.6%)	**.038**
Pure TEVAR	7 (9.3%)	10 (11.2%)	17 (10.4%)	.800
Follow-up status				
Follow-up index (imaging/CTS visit)	0.5 ± 0.4	0.7 ± 0.4	0.6 ± 0.4	**.023**
Follow-up index (survival)	0.7 ± 0.4	0.8 ± 0.3	0.7 ± 0.3	.060

Numeric variables are shown as mean ± SD. Categorical variables are shown as N (%). Bold values denote *P* < .05. *BMI*, Body mass index; *AVR*, aortic valve replacement; *TEVAR*, thoracic endovascular aortic repair; *OR*, operating room; *CTS*, cardiothoracic surgery.

The reintervention rate was significantly lower in RR compared with non-RR (17.3% vs 38.2%, *P =* .003). RR had significantly lower reoperation at the root (4% vs 18%, *P =* .006), at the arch (6.7% vs 20.2%, *P =* .014), and distal to the arch (14.7% vs 29.2%, *P =* .038). Late mortality was similar (6.7% vs 9.0%, *P =* .773). The postoperative outcomes remained similar after propensity score matching for age, gender, and preoperative aneurysm.

**Figure 2:**
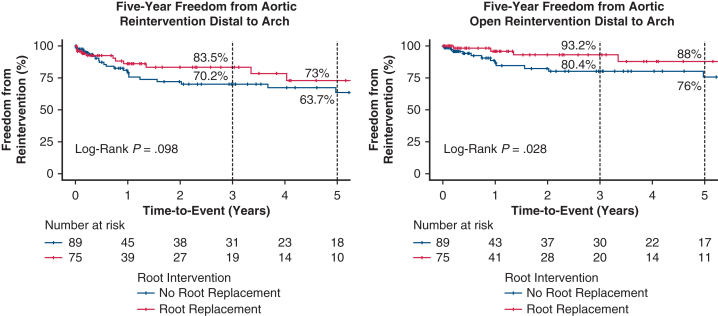
*Left*: comparison of 5-year freedom from distal reintervention between RR and non-RR. *Right*: comparison of 5-year freedom from open distal reintervention between RR and non-RR. Maximum follow-up years: RR – 11.2 years, non-RR – 11.1 years. *RR*, Root replacement; *non-RR*, non-root replacement.

**Figure E1:**
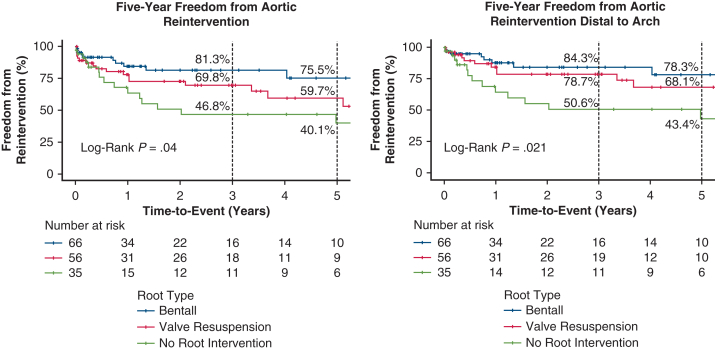
*Left*: comparison of 5-year freedom from any reintervention among different types of root intervention. Pairwise comparisons: Bentall versus valve resuspension (p_logrank_ = .184), Bentall versus no root intervention (p_logrank_ = .042), valve resuspension versus no root intervention (p_logrank_ = .184). *Right*: comparison of 5-year freedom from reintervention distal reintervention among different types of root intervention. Pairwise comparisons: Bentall versus valve resuspension (p_logrank_ = .225), Bentall versus no root intervention (p_logrank_ = .031), valve resuspension versus no root intervention (p_logrank_ = .156). Maximum follow-up years: Bentall – 11.2 years, valve resuspension – 11.1 years, no root intervention – 8.7 years.

Additionally, RR demonstrated significantly higher 5-year freedom from any reintervention compared with non-RR (70.7% vs 56.9%, p_logrank_ = .029). Although the 5-year freedom from distal reintervention was not significantly different (73% vs 63.7%, p_logrank_ = .098), the 5-year freedom from open distal reintervention was better in the RR compared with the non-RR group (88% vs 76%, p_logrank_ = .028) (Figure 2). When performed by the high-volume surgeon, RR had higher 5-year freedom from any distal reintervention (87.1% vs 72.8%, p_logrank_ = .033). To further analyze different root interventions, the 2 groups were stratified into 5 subgroups: Bentall procedure (n = 66), David procedure (n = 5), valve resuspension with root repair/remodeling (n = 56), root reinforcement only (n = 2), and no root intervention (n = 35). Because of the extremely small numbers undergoing the David procedure and root reinforcement only, these subgroups were excluded from the Kaplan–Meier analysis (Figure E1). Pairwise comparisons showed similar 5-year freedom from any reintervention between Bentall procedures and valve resuspensions (75.5% vs 59.7%, p_logrank_ = .184). Bentall procedures, however, showed higher freedom from any reintervention compared with no root intervention (75.5% vs 40.1%, p_logrank_ = .042). This pattern was also observed for distal reintervention (p_logrank_ = .225 and p_logrank_ = .031, respectively).

### Extent of Arch Intervention

Among 164 patients, 97 (59.1%) underwent HAR, 54 (32.9%) received TAR, and 13 (7.9%) had no arch intervention (non-AR, ascending or root only). No significant differences were observed in demographics and comorbidities among the groups. HAR (88.7%) and TAR (74.1%) were more commonly performed emergently compared with non-AR (61.5%, *P =* .010) (Table 2).

**Table 2:** Comparisons of preoperative, operative, and postoperative variables among hemiarch, total arch replacement, and non-arch replacement (ascending or root replacement only)

Variable	Hemiarch (N = 97)	Total arch (N = 54)	Ascending or root only (N = 13)	*P* value
Patient demographics and comorbidities				
Age, y	60.1 ± 13.3	57 ± 12.2	60.9 ± 14.2	.335
Sex (male)	65 (67%)	42 (77.8%)	6 (46.2%)	.071
BMI	28.5 ± 5.1	29.4 ± 5.9	25.6 ± 4.7	.113
Hypertension	72 (74.2%)	46 (85.2%)	11 (84.6%)	.262
Smoking	24 (24.7%)	20 (37%)	6 (46.2%)	.115
Connective tissue disorder	4 (4.1%)	1 (1.9%)	0 (0.0%)	.773
Presentation				
Urgency status (emergency)	86 (88.7%)	40 (74.1%)	8 (61.5%)	**.010**
Extent of dissection	-	-	-	-
Root	50 (51.5%)	38 (70.4%)	4 (30.8%)	**.014**
Ascending	91 (93.8%)	47 (87%)	13 (100%)	.258
Arch	62 (63.9%)	52 (96.3%)	10 (76.9%)	**<.001**
Descending	23 (23.7%)	38 (70.4%)	5 (38.5%)	**<.001**
Preoperative hemodynamic instability	34 (35.1%)	22 (40.7%)	3 (23.1%)	.496
Cardiogenic shock	17 (17.5%)	3 (5.6%)	1 (7.7%)	.102
Hypertensive crisis	4 (4.1%)	2 (3.7%)	0 (0.0%)	1.000
Massive transfusion protocol	0 (0.0%)	0 (0.0%)	0 (0.0%)	1.000
Intubation	2 (2.1%)	2 (3.7%)	1 (7.7%)	.336
Mechanical circulatory support	1 (1%)	0 (0.0%)	0 (0.0%)	1.000
Malperfusion	18 (18.6%)	21 (38.9%)	1 (7.7%)	**.008**
Aneurysm	27 (27.8%)	22 (40.7%)	5 (38.5%)	.238
Other operative interventions				
AVR	46 (47.4%)	25 (46.3%)	1 (7.7%)	**.018**
Root	46 (47.4%)	28 (51.9%)	1 (7.7%)	**.010**
Ascending	93 (95.9%)	51 (94.4%)	12 (92.3%)	.614
TEVAR	13 (13.4%)	50 (92.6%)	1 (7.7%)	**<.001**
Postoperative outcomes				
Time from OR to death (y)	5.7 ± 3.0	1.5 ± 1.6	0.9 ± 0.2	**.017**
Late death (beyond 30 d postoperatively)	6 (6.2%)	5 (9.3%)	2 (15.4%)	.336
Stable ≥18-mo postoperative imaging	38 (80.9%)	22 (88%)	4 (50%)	.065
Time to last follow-up or imaging if no surgical intervention (y)	3.0 ± 3.3	2.3 ± 2.2	4.4 ± 5.8	.647
Time to first reoperation (y)	2.3 ± 3.0	0.4 ± 0.5	2.4 ± 2.8	**.015**
Reoperation	23 (23.7%)	14 (25.9%)	10 (76.9%)	**.001**
Valve	9 (9.3%)	4 (7.5%)	3 (23.1%)	.231
Root	10 (10.3%)	5 (9.3%)	4 (30.8%)	.101
Ascending	16 (16.5%)	1 (1.9%)	3 (23.1%)	**.005**
Distal to root	20 (20.6%)	10 (18.5%)	9 (69.2%)	**.001**
Arch	18 (18.6%)	1 (1.9%)	4 (30.8%)	**.001**
Distal to arch	19 (19.6%)	10 (18.5%)	8 (61.5%)	**.005**
Distal to arch (open)	15 (15.5%)	1 (1.9%)	4 (30.8%)	**.002**
Descending	19 (19.6%)	10 (18.5%)	8 (61.5%)	**.005**
Pure TEVAR	4 (4.1%)	9 (16.7%)	4 (30.8%)	**.002**
Follow-up status				
Follow-up index (imaging/CTS visit)	0.6 ± 0.4	0.6 ± 0.4	0.8 ± 0.4	.059
Follow-up index (survival)	0.8 ± 0.3	0.7 ± 0.3	0.6 ± 0.4	.180

Numeric variables are shown as mean ± SD. Categorical variables are shown as N (%). Bold values denote *P* < .05. *BMI*, Body mass index; *AVR*, aortic valve replacement; *TEVAR*, thoracic endovascular aortic repair; *OR*, operating room; *CTS*, cardiothoracic surgery.

TAR had more preoperative malperfusion than HAR and non-AR (38.9% vs 18.6% vs 7.7%, *P =* .008). HAR showed a higher rate of preoperative cardiogenic shock than TAR (17.5% vs 5.6%, *P =* .045). Differences were also observed in the extent of dissection. Approximately half of the HAR and TAR cases involved concomitant AVR and RR. FET was used in 92.6% of total arch cases.

Significant differences were seen in any reintervention (*P =* .001), arch reintervention (*P =* .001), and reintervention distal to arch (*P =* .005) among the 3 groups. Arch reoperation occurred in 1 patient (1.9%) after TAR, compared with 18.6% for HAR and 30.8% for non-AR. However, pairwise comparisons between HAR and TAR found no significant differences in distal reintervention. Notably, 90% of distal reinterventions in TAR were performed with pure TEVAR. Furthermore, TAR had significantly fewer open redo distal to the arch compared with HAR (1.9% vs 15.5%, *P =* .011).

**Figure 3:**
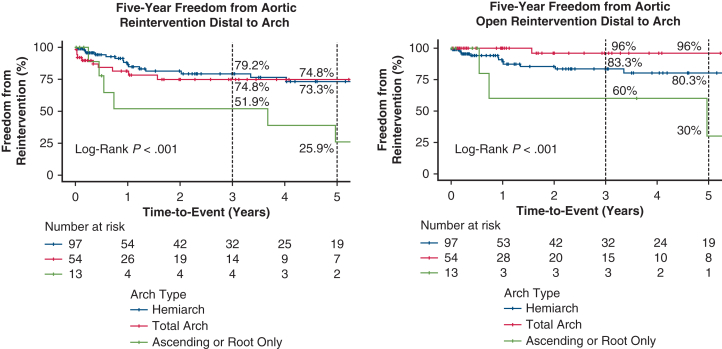
*Left*: comparison of 5-year freedom from distal reintervention among hemiarch, total arch, and no arch replacements. Pairwise comparisons: hemiarch versus total arch (p_logrank_ = .463), hemiarch versus ascending or root only (p_logrank_ < .001), total arch versus ascending or root only (p_logrank_ = .019). *Right*: comparison of 5-year freedom from open distal reintervention among hemiarch, total arch, and no arch replacements. Pairwise comparisons: hemiarch versus total arch (p_logrank_ = .048), hemiarch versus ascending or root only (p_logrank_ = .010), total arch versus ascending or root only (p_logrank_ < .001). Maximum follow-up years: hemiarch – 11.2 years, total arch – 7.2 years, ascending or root only – 6.7 years.

Both TAR and HAR demonstrated improved 5-year freedom from any reintervention compared with non-AR (70.3% vs 21.6%, p_logrank_ = .022, 67% vs 21.6%, p_logrank_ < .001, respectively). Similar benefits were observed in 5-year freedom from root reintervention (86.4% vs 53.3%, p_logrank_ = .026, 87.9% vs 53.3%, p_logrank_ = .010, respectively), reintervention distal to the arch (74.8% vs 25.9%, p_logrank_ = .019, 73.3% vs 25.9%, p_logrank_ < .001, respectively), and open distal reintervention (96% vs 30%, p_logrank_ < .001, 80.3% vs 30%, p_logrank_ = .010, respectively) (Figure 3). TAR had improved 5-year freedom from open distal reintervention compared with HAR (96% vs 80.3%, p_logrank_ = .048).

### Root Replacement With Different Types of Arch Intervention Versus Non-Root Replacement

The root replacement group was stratified into root with hemiarch replacement (RR-HAR, n = 46, 28.0%), root with total arch replacement (RR-TAR, n = 28, 17.1%), and root without arch replacement (n = 1, 0.6%) subgroups (Table E1). Because of the single patient in the root without arch replacement subgroup, further analysis for this subgroup was not performed.

Age was higher in the non-RR group compared with the RR-HAR and RR-TAR groups (*P =* .007). Preoperative aneurysm was more frequent in the RR-TAR group than in the RR-HAR and non-RR groups (53.6% vs 37.0% vs 24.7%, *P =* .016). No differences were seen in BMI, other comorbidities, or urgency status. The extent of dissection varied among the subgroups. Operative techniques varied significantly, whereas imaging stability, time to death, and time to first reoperation showed no significant differences.

The overall reintervention rate was significantly different among the subgroups (*P =* .013). RR-HAR showed a rate of 15.2%, compared with 38.2% for non-RR and 21.4% for RR-TAR. Arch reoperation (*P =* .012) and open redo distal to the arch (*P =* .020) were also significantly different, but overall reoperation distal to the arch was not (*P =* .100). Notably, in RR-TAR, the distal reoperations (17.9%) were all endovascular repairs. Late mortality rates were similar across subgroups (*P =* .925).

**Figure 4:**
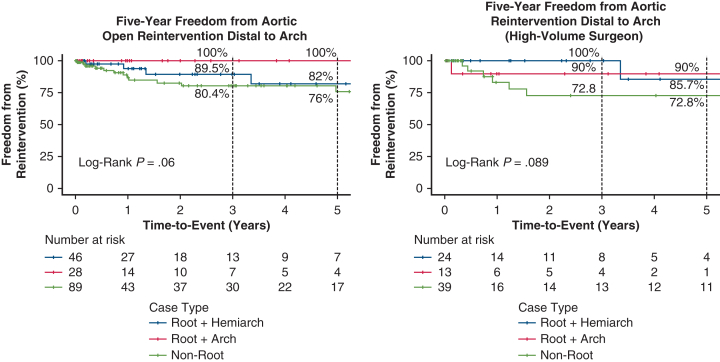
*Left*: comparison of 5-year freedom from open distal reintervention among combined root and HARs, combined root and TARs, and non-RRs. Pairwise comparisons: root + hemiarch versus root + total arch (p_logrank_ = .149), root + hemiarch versus non-root (p_logrank_ = .149), root + total arch versus non-root only (p_logrank_ = .149). Maximum follow-up years: root + hemiarch – 11.2 years, root + total arch – 7.2 years, non-root – 11.1 years. *Right*: comparison of 5-year freedom from overall distal reintervention among combined root and HARs, combined root and TARs, and non-RRs, when operated by the high-volume surgeon. Pairwise comparisons: root + hemiarch versus root + total arch (p_logrank_ = .564), root + hemiarch versus non-root (p_logrank_ = .091), root + total arch versus non-root only (p_logrank_ = .564). Maximum follow-up years: root + hemiarch – 11.2 years, root + total arch – 5.5 years, non-root – 11.1 years. *HAR*, Hemiarch replacement; *TAR*, total arch replacement; *non-RR*, non-root replacement.

Five-year freedom from any reintervention was highest in RR-TAR (75.2%), followed by RR-HAR (69.6%), and non-RR (56.9%), although the differences were not statistically significant (p_logrank_ = .060). Likewise, no significant difference was observed in 5-year freedom from distal reintervention among all subgroups (p_logrank_ = .170). A further analysis (Figure 4) on 5-year freedom from open distal reintervention found a marginal difference: RR-TAR: 100%, RR-HAR 82%, and non-RR 76% (p_logrank_ = .060).

In contrast, when performed by the high-volume surgeon, a significant difference was observed in 5-year freedom from any reintervention (p_logrank_ = .030) with a borderline difference in 5-year freedom from distal reintervention (p_logrank_ = .089) (Figure 4).

### Risk Predictors for Reinterventions

#### Any reintervention

RR showed a 50.4% risk reduction in the univariate analysis (hazard ratio [HR], 0.496, CI, 0.261-0.943, *P =* .032) but did not reach significance in the multivariate model (HR, 0.623, CI, 0.318-1.222, *P =* .169). HAR was protective in both univariate (HR, 0.452, CI, 0.249-0.819, *P =* .009) and multivariate analyses (HR, 0.282, CI, 0.126-0.633, *P =* .002) (Table 3). TAR demonstrated a trend toward risk reduction in the multivariate model (HR, 0.425, CI, 0.175-1.030, *P =* .058). Neither distal dissection (arch and descending aorta) nor high-volume surgeon was a significant predictor of any reintervention over the follow-up period.

**Table 3:** Cox regression model results showing risk factors for any reintervention and reintervention distal to the arch over the course of the long-term follow-up period in this study

Variable	Univariate analysis			Multivariate analysis		
	HR	95% CI	*P* value	HR	95% CI	*P* value
a. Cox regression – any reintervention						
Arch/descending dissection	1.532	0.740-3.171	.25	1.261	0.584-2.722	.554
High-volume surgeon	0.556	0.300-1.033	.063	0.72	0.378-1.372	.318
Root replacement	0.496	0.261-0.943	**.032**	0.623	0.318-1.222	.169
Hemiarch	0.452	0.249-0.819	**.009**	0.282	0.126-0.633	**.002**
Total arch	1.178	0.623-2.227	.614	0.425	0.175-1.030	.058
b. Cox regression – reintervention distal to arch (both open and endovascular)						
Arch/descending dissection	1.649	0.723-3.761	.234	1.389	0.584-3.301	.457
High-volume surgeon	0.415	0.199-0.864	**.019**	0.534	0.249-1.145	.107
Root replacement	0.553	0.272-1.127	.103	0.723	0.342-1.528	.395
Hemiarch	0.484	0.246-0.951	**.035**	0.295	0.119-0.732	**.008**
Total arch	1.040	0.496-2.180	.917	0.381	0.138-1.053	.063
c. Cox regression – reintervention distal to arch (open)						
Arch/descending dissection	1.555	0.518-4.672	.432	-	-	-
High-volume surgeon	0.485	0.183-1.289	.147	-	-	-
Root replacement	0.307	0.102-0.930	**.037**	0.391	0.125-1.226	.107
Hemiarch	1.349	0.474-3.839	.574	0.315	0.098-1.010	.052
Total arch	0.139	0.018-1.048	.056	0.056	0.006-0.518	**.011**

a: Any reintervention. b: Overall distal reintervention (both open and endovascular). c: Open distal reintervention only. Arch/descending dissection and high-volume surgeon were not included in the multivariate analysis to avoid model overfitting. Bold values denote *P* < .05. *HR*, Hazard ratio.

#### Overall distal reintervention

High-volume surgeons significantly reduced the risk of reintervention distal to the arch in univariate analysis (HR, 0.415, CI, 0.199-0.864, *P =* .019), but not in multivariate analysis. HAR was protective in both univariate (HR, 0.484, CI, 0.246-0.951, *P =* .035) and multivariate analyses (HR, 0.295, CI, 0.119-0.732, *P =* .008). TAR also showed a trend toward protection in adjusted analysis (HR, 0.381, 95% CI, 0.138-1.053, *P =* .063).

#### Open distal reintervention

A subanalysis of open reinterventions distal to the arch (excluding pure TEVAR) revealed a 69.3% reduction in risk with RR in the univariate model (HR, 0.307, CI, 0.102-0.930, *P =* .037). In multivariate analysis, HAR was marginally protective (HR, 0.315, CI, 0.098-1.010, *P =* .052), and TAR significantly reduced risk by 94.4% (HR, 0.056, CI, 0.006-0.518, *P =* .011).

## Discussion

The present study evaluated the long-term outcomes (eg, reintervention and survival) of different root and arch management strategies in patients with ATAAD. It highlighted the importance of careful selection of operative plans because they significantly impacted freedom from reintervention and overall repair durability.

### Root Intervention

Conservative approaches such as valve resuspension, reapproximation of dissected wall, and neo-reconstruction are relatively reproducible by low-volume surgeons, but raise concern for long-term durability because the pathologic native root can continue to dilate and further dissect. On the other hand, more extensive repairs such as RR with or without valve replacement (VSRR) are reasonable options that can be safely performed at high-volume centers, although they may have increased short-term risks.^18^, ^19^, ^20^ Existing literature including a large-scale International Registry of Acute Aortic Dissection study reported similar perioperative mortality, midterm survival, and freedom from root reintervention when comparing RR and more conservative root managements.^14^^,^^21^, ^22^, ^23^ However, some studies showed that the reoperation risk was higher with limited root repair or supracommissural replacement compared with non-valve-preserving root replacement.^23^, ^24^, ^25^, ^26^, ^27^ The current literature has focused primarily on comparing the freedom from proximal reintervention of conservative versus more extensive approaches. VSRR, for dissections and aneurysms, was reported to have a 5.1% cumulative incidence of distal reintervention at 10 years.^28^ For ATAAD alone, the impact of root strategy on distal aortic pathology and reintervention remained unclear.

This study showed that patients undergoing RR had better long-term freedom from any reintervention and distal reintervention, particularly when operated by the high-volume surgeon, compared with non-RR. In univariate Cox regression analysis, RR demonstrated a 50.4% and 69.3% reduction in risk for any reintervention and open reintervention distal to the arch, respectively. In subanalysis, the Bentall procedure showed improved 5-year freedom from any and distal reinterventions compared with those with only valve resuspension or no root intervention. These findings suggest that RR, despite being more technically challenging, provides a more durable solution, particularly in preventing distal reinterventions.

The authors propose 2 hypotheses to explain the protective effect of RR on distal reintervention. First, synthetic root grafts may better withstand high blood pressure compared with native, repaired, or nonrepaired aortic tissues, thereby stabilizing the dissection, preventing propagation, and improving hemodynamic flow. Second, RR prevents re-dissection into the root and further extension of the dissection. In this cohort, RR was frequently combined with AVR (86.7%), potentially contributing to its protective effects. Almost all RR patients (98.7%) underwent HAR or TAR, suggesting the protective effects may be partly due to arch intervention. This aligns with multivariate Cox regression, which showed RR may not independently protect against distal reintervention.

### Arch Intervention

HAR is the standard of care for ATAAD^2^^,^^29^ and is recommended in limited-resource or low-volume centers. However, TAR is favored by some surgeons due to concerns about distal complications.^15^^,^^16^ A German registry study indicated that hemiarch had a trend toward lower mortality and a comparable rate of new neurological deficits when compared with TAR.^30^ However, a higher stroke rate and perioperative mortality were observed in patients receiving TAR according to Society of Thoracic Surgeons database studies.^31^ Interestingly, although there is evidence of cerebral malperfusion, TAR with carotid artery replacement appears to have a lower rate of stroke compared with HAR.^32^ Furthermore, there have been reports of using TAR with FET to treat malperfusion caused by the false lumen, especially in abdominal viscera.^12^ From a pathophysiological standpoint, HAR is associated with a longer suture line leading to concern for suture cutting during the early postoperative period and unfavorable distal remodeling in the later phase, whereas TAR has a more secured anastomosis and favorable distal remodeling afterward.^33^ A meta-analysis comparing HAR and TAR reported similar in-hospital mortality, long-term survival, and long-term freedom from reoperation; however, these original results were derived from studies conducted at high-volume centers.^34^ A more recent meta-analysis showed better long-term survival and comparable reoperation rate (lower in TAR when >7 years).^35^ According to existing literature, multiple factors should be taken into consideration when planning on arch intervention.

To date, there have been few articles focusing on distal reoperation after ATAAD repair. The present study explored the role of arch interventions in the long-term outcomes of ATAAD. It evaluated reinterventions, focusing on those involving the root, arch, and distal to the arch. The most common indications for reoperation beyond the root were for residual or new dissections and new or growing aneurysms in the arch or descending thoracic aorta. In the Cox regression models, although HAR was superior in protecting against overall distal reinterventions (including open and endovascular procedures), it had only a borderline protective effect against open distal reintervention. In contrast, TAR, despite showing marginal protective benefits against overall distal reintervention, significantly reduced the risk of open distal reintervention. This suggests that TAR during initial ATAAD repair may prevent high-risk open reinterventions, which are associated with greater costs and risks compared with TEVAR, thereby improving long-term outcomes.

When stratifying patients by both root and arch interventions, Kaplan–Meier analysis showed root with total arch replacement performed by the high-volume surgeon was associated with borderline improved freedom from overall distal reintervention at 5 years compared with non-root replacement. There was no significant difference between root combined with total arch versus root combined with hemiarch, suggesting both were reasonable approaches for ATAAD when indicated. All distal reinterventions in the root combined with total arch group were performed with TEVAR, although whether these were planned or unplanned was not explicitly documented. The shorter time to first reoperation in this subgroup likely reflects planned interval TEVAR extensions. Notably, the complete absence of open distal reinterventions in root with TAR underscored the effectiveness in preventing the risks associated with redo open surgery. Similar long-term survival among all subgroups supports performing TAR in suitable patients.

Interestingly, arch intervention, whether hemiarch or TAR, appeared to be protective against root reintervention in Kaplan–Meier analysis. The authors propose 2 biological mechanisms. Both hemiarch and TAR stabilize the proximal aspect of the arch and often the ascending aorta. Eliminating dissection within the ascending aorta/arch is crucial, because it prevents residual dissection from retrograde propagation into the root. A replaced hemiarch or entire arch also may improve hemodynamic flow and reduce turbulence, which could have contributed to the initial dissection, thereby lowering the risk of new tears in the root. Additionally, approximately half of the hemiarch and TAR patients in this cohort also underwent simultaneous RR, potentially confounding the observed risk reduction for root reintervention.

### High-Volume Surgeon

**Figure E2:**
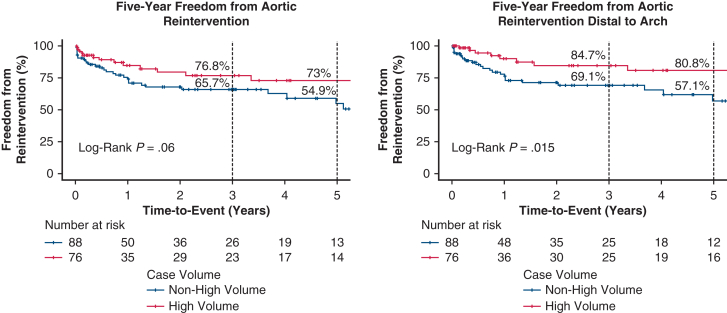
*Left*: comparison of 5-year freedom from any reintervention between high-volume and non–high-volume surgeons. *Right*: comparison of 5-year freedom from reintervention distal reintervention high-volume and non–high-volume surgeons. Maximum follow-up years: high-volume – 11.2 years, non–high-volume – 11.0 years.

Kaplan–Meier analysis found that high case volume had significantly higher freedom from any reintervention and distal reintervention (Figure E2). Although high-volume surgeon was protective against distal reintervention in the univariate analysis, the benefit was not observed in the multivariate analysis. This finding suggests that although the surgeon's experience may provide advantages, successful outcomes can be achieved primarily by focusing on the type and quality of interventions for ATAAD, regardless of the surgeon's volume.

### Others

This study also evaluated the imaging stability across different postoperative time points. No differences were observed among the various root and arch approaches in patients with available imaging data. The likely explanation is the low sample sizes of available imaging results. Additionally, some patients in this cohort were from out of state or had postoperative care and follow-up at different institutes, which may not adhere to the same imaging protocol, potentially impacting dissection surveillance consistency. This emphasizes the importance of standardized dissection surveillance protocols to ensure consistent and accurate long-term monitoring.

### Limitations

- This is a retrospective, single-center study.
- Surgical decisions were based on the stated criteria, but individual surgeon preferences or experience could not be assessed.
- Loss to follow-up rates were 19.8%, 32.1%, and 46.8% at 1 month, 3 months, and 1 year, respectively. Reintervention rates were likely under-reported due to the retrospective design and follow-up losses, primarily caused by out-of-network insurance or patients living out of state.
- Other preoperative comorbidities (eg, hyperlipidemia, coronary artery disease, stroke) were not included in this study.
- Short-term postoperative outcomes and complications were beyond the scope of this study and not compared.
- Small sample sizes after stratification and moderate follow-up loss may have limited statistical power, potentially underestimating subgroup differences.
- It was unclear whether TEVAR extensions were planned or unplanned due to the retrospective design. Therefore, the authors specifically analyzed open distal reoperations to eliminate this uncertainty.
- To minimize confounding, 30-day mortality cases were excluded. A comparison of patient characteristics between those who survived more than 30 days and those who died within 30 days can be found in Table E2. However, root and arch replacements may impose a higher perioperative burden due to the complexity of repair, although they were performed when clinically indicated for patients with more extensive dissection and hemodynamic instability.

## Conclusions

These data suggest more aggressive approaches in terms of root and arch replacement in patients with ATAAD could be protective. The significant reduction in the reoperation rates associated with these approaches demonstrates their potential to reduce the burden and cost of subsequent operations and improve long-term outcomes. However, although RR and TAR offer excellent long-term benefits, these procedures are more complex and potentially associated with higher immediate perioperative risks. The decision to pursue these more extensive interventions should be based on a comprehensive patient assessment, including the extent of aortic dissection, the presence of any comorbidities, and the surgeon's optimal approach.

## Conflict of Interest Statement

The authors reported no conflicts of interest.

The *Journal* policy requires editors and reviewers to disclose conflicts of interest and to decline handling or reviewing manuscripts for which they may have a conflict of interest. The editors and reviewers of this article have no conflicts of interest.

## Figures and Tables

**Table E1:** Comparisons of preoperative, operative, and postoperative variables among root replacement with hemiarch and total arch replacements, and non-root replacement

Variable	Root + hemiarch (N = 46)	Root + arch (N = 28)	Non-root (N = 89)	*P* value
Patient demographics and comorbidities				
Age, y	58.5 ± 13.6	52.8 ± 11.3	61.5 ± 12.7	**.007**
Sex (male)	35 (76.1%)	23 (82.1%)	55 (61.8%)	.070
BMI	28.2 ± 5.3	29.2 ± 5.5	28.5 ± 5.5	.762
Hypertension	34 (73.9%)	24 (85.7%)	70 (78.7%)	.496
Smoking	11 (23.9%)	8 (28.6%)	30 (33.7%)	.525
Connective tissue disorder	2 (4.3%)	1 (3.6%)	2 (2.2%)	.707
Presentation				
Urgency status (emergency)	41 (89.1%)	21 (75%)	71 (79.8%)	.251
Extent of dissection	-	-	-	-
Root	34 (73.9%)	26 (92.9%)	31 (34.8%)	**<.001**
Ascending	40 (87%)	28 (100%)	82 (92.1%)	.130
Arch	25 (54.3%)	26 (92.9%)	72 (80.9%)	**<.001**
Descending	11 (23.9%)	20 (71.4%)	34 (38.2%)	**<.001**
Preoperative hemodynamic instability	16 (34.8%)	13 (46.4%)	30 (33.7%)	.487
Cardiogenic shock	10 (21.7%)	3 (10.7%)	8 (9%)	.116
Hypertensive crisis	1 (2.2%)	1 (3.6%)	4 (4.5%)	.861
Massive transfusion protocol	0 (0.0%)	0 (0.0%)	0 (0.0%)	1.000
Intubation	1 (2.2%)	2 (7.1%)	2 (2.2%)	.381
Mechanical circulatory support	0 (0.0%)	0 (0.0%)	1 (1.1%)	1.000
Malperfusion	8 (17.4%)	10 (35.7%)	22 (24.7%)	.226
Aneurysm	17 (37%)	15 (53.6%)	22 (24.7%)	**.016**
Other operative interventions				
AVR	39 (84.8%)	25 (89.3%)	7 (7.9%)	**<.001**
Ascending	43 (93.5%)	26 (92.9%)	87 (97.8%)	.312
TEVAR	5 (10.9%)	27 (96.4%)	32 (36%)	**<.001**
Postoperative outcomes				
Time from OR to death (y)	6.8 ± 4.4	0.6 ± 0.4	2.7 ± 2.0	.089
Late death (beyond 30 d postoperatively)	3 (6.5%)	2 (7.1%)	8 (9%)	.925
Stable ≥18-mo postoperative imaging	17 (85%)	12 (100%)	35 (72.9%)	.075
Time to last follow-up or imaging if no surgical intervention (y)	2.5 ± 2.9	2.0 ± 2.2	3.4 ± 3.6	.349
Time to first reoperation (y)	1.5 ± 1.5	0.2 ± 0.3	2.1 ± 2.9	**.024**
Reoperation	7 (15.2%)	6 (21.4%)	34 (38.2%)	**.013**
Valve	1 (2.2%)	0 (0.0%)	15 (16.9%)	**.003**
Root	2 (4.3%)	1 (3.6%)	16 (18%)	**.024**
Ascending	4 (8.7%)	0 (0.0%)	16 (18%)	**.020**
Distal to root	6 (13%)	5 (17.9%)	28 (31.5%)	**.045**
Arch	5 (10.9%)	0 (0.0%)	18 (20.2%)	**.012**
Distal to arch	6 (13%)	5 (17.9%)	26 (29.2%)	.100
Distal to arch (open)	4 (8.7%)	0 (0.0%)	16 (18%)	**.020**
Descending	6 (13%)	5 (17.9%)	26 (29.2%)	.100
Pure TEVAR	2 (4.3%)	5 (17.9%)	10 (11.2%)	.165
Follow-up status				
Follow-up index (imaging/CTS visit)	0.5 ± 0.4	0.6 ± 0.4	0.7 ± 0.4	.084
Follow-up index (survival)	0.7 ± 0.3	0.7 ± 0.4	0.8 ± 0.3	.193

Numeric variables are shown as mean ± SD. Categorical variables are shown as N (%). Bold values denote *P* < .05. *BMI*, Body mass index; *AVR*, aortic valve replacement; *TEVAR*, thoracic endovascular aortic repair; *OR*, operating room; *CTS*, cardiothoracic surgery.

**Table E2:** Comparisons of preoperative, operative, and postoperative variables between those who survived beyond 30 days and those who died within 30 days

Variable	Survivors (>30 d) (N = 177)	30-d mortality (N = 35)	Overall (N = 212)	*P* value
Patient demographics and comorbidities				
Age, y	59.7 ± 13.5	57.4 ± 11.4	59.3 ± 13.2	.306
Sex (male)	122 (68.93%)	23 (65.71%)	149 (68.98%)	.695
BMI	28.6 ± 5.6	29.9 ± 8.9	28.8 ± 6.2	.795
Hypertension	141 (79.66%)	26 (74.29%)	170 (78.7%)	.500
Smoking	54 (30.51%)	12 (34.29%)	68 (31.48%)	.692
Connective tissue disorder	5 (2.82%)	1 (3.57%)	6 (2.87%)	.591
Presentation				
Urgency status (emergency)	144 (81.36%)	35 (100%)	182 (84.26%)	**.002**
Extent of dissection	-	-	-	-
Root	97 (54.8%)	25 (71.43%)	123 (56.94%)	.092
Ascending	160 (90.4%)	33 (94.29%)	197 (91.2%)	.746
Arch	130 (73.45%)	31 (88.57%)	164 (75.93%)	.081
Descending	70 (39.55%)	22 (62.86%)	92 (42.59%)	**.015**
Preoperative hemodynamic instability	61 (35.26%)	26 (74.29%)	89 (41.98%)	**<.001**
Cardiogenic shock	22 (12.72%)	9 (25.71%)	31 (14.62%)	.066
Hypertensive crisis	7 (4.05%)	3 (8.57%)	10 (4.72%)	.377
Massive transfusion protocol	0 (0.0%)	2 (5.71%)	2 (0.94%)	**.028**
Intubation	5 (2.89%)	5 (14.29%)	10 (4.72%)	**.014**
Mechanical circulatory support	1 (0.58%)	0 (0.0%)	1 (0.47%)	1.000
Malperfusion	41 (23.7%)	24 (68.57%)	67 (31.6%)	**<.001**
Aneurysm	55 (31.79%)	11 (31.43%)	67 (31.6%)	1.000
Operative interventions				
AVR	77 (43.5%)	24 (68.57%)	104 (48.15%)	**.009**
Root	81 (45.76%)	23 (65.71%)	106 (49.07%)	**.041**
Ascending	169 (95.48%)	34 (97.14%)	207 (95.83%)	1.000
Hemiarch	109 (61.58%)	11 (31.43%)	123 (56.94%)	**.001**
Total arch	55 (31.07%)	22 (62.86%)	77 (35.65%)	**.001**
TEVAR	66 (37.29%)	21 (60%)	88 (40.74%)	**.015**

Absence of postoperative imaging was not an exclusion criterium. Numeric variables are shown as mean ± SD. Categorical variables are shown as N (%). Bold values denote *P* < .05. *BMI*, Body mass index; *AVR*, aortic valve replacement; *TEVAR*, thoracic endovascular aortic repair.
